# Case report on a swift shift in uropathogens from *Shigella flexneri* to *Escherichia coli*: a thin line between bacterial persistence and reinfection

**DOI:** 10.1186/s12941-020-00374-y

**Published:** 2020-07-29

**Authors:** Kukwah Anthony Tufon, Djike Puepi Yolande Fokam, Youmbi Sylvain Kouanou, Henry Dilonga Meriki

**Affiliations:** 1 Buea Regional Hospital, Southwest Region, Buea, Cameroon; 2 Department of Allied Health, Faculty of Health Science, Biaka University, Buea, Cameroon; 3 Department of Microbiology and Parasitology, Faculty of Science, University of Buea, Buea, Cameroon; 4 Department of Internal Medicine and Paediatrics, Faculty of Health Science, University of Buea, Buea, Cameroon

**Keywords:** Urinary tract infection, *Escherichia coli*, *Shigella flexneri*, Reinfection, Urine culture

## Abstract

**Background:**

Urinary tract infections (UTI) are mostly caused by bacteria. Urine cultures are usually a definitive measure to select the appropriate antibiotics for the elimination of a uropathogen and subsequent recovery from the infection. However, the preferred antibiotics as determined by urine culture and sensitivity may still not eliminate the infection and would require further examination to ascertain the cause of treatment failure which could be unresolved bacteriuria, bacterial persistence, immediate reinfection with a different uropathogen or misdiagnosis.

**Case presentation:**

A 2-years 7 months-old female was admitted in the Regional hospital of Buea following persistent fever. An auto medication with amoxicillin was reported. Urinalysis was done on the first day and the sediment of the cloudy urine revealed many bacteria and few pus cells. Ceftriaxone was prescribed as empirical treatment and a request for urine and blood culture was made. Three days after admission, the temperature and CRP were 39.0 °C and 96 mg/l, respectively. The urine culture results (> 10^5^ CFU/ml of *Shigella flexneri* sensitive to ofloxacin) were presented to the doctor on the 4th day of admission. Patient was put on ofloxacin. Three days after, the temperature (38.5 °C) and CRP (24 mg/l) were still elevated. The blood culture result came out negative. A second urine culture was requested which came back positive (> 10^5^ CFU/ml of *Escherichia coli* resistant to ofloxacin and sensitive to meropenem and amikacin). Ofloxacin was discontinued and the patient put on meropenem and amikacin. The third urine culture recorded no significant growth after 48 h of incubation. The patient was discharged looking healthy once more with a normal body temperature.

**Conclusion:**

Antibiotics tailored towards the elimination of a particular bacterial species may as well provide a favorable environment for other bacterial species that are resistant to it in the course of treating a UTI episode. This apparent treatment failure may first of all require a second urine culture for confirmation rather than considering the possibilities of a misdiagnosis.

## Background

Urinary tract infection (UTI) annually affects about 150 million people globally [1]. Although other microorganisms have been reported to cause UTI, it is mostly caused by bacteria and usually treated with antibiotics [2]. Most common uropathogens include *Escherichia coli*, *Klebsiella pneumoniae*, *Proteus Mirabilis*, *Enterococcus* sp., *Staphylococcus saprophyticus*, and *Pseudomonas aeruginosa* [3, 4]. UTI caused by *Shigella* sp. have been identified in rare occasions [5–8].

Although contamination is usually considered when more than one bacterial species is isolated from urine, some UTI are actually caused by 2 bacterial species acting simultaneously [9, 10]. However, a rapid and successive shift from one bacterial species to another (following antimicrobial therapy) in the course of a particular UTI episode has not been reported before to the best of our knowledge. An understanding on how to identify and manage such infections is of paramount importance in making clinical decisions for patients with complicated UTI.

## Case presentation

A 2 years 7 months-old female with a body mass of 12.8 kg was admitted in the Regional hospital of Buea on the 31st of March 2019 after a 4-day persistent fever, polyarthralgia and abdominal pain but no vomiting nor diarrhea. The patient who lives in an urban area with access to drinking water had no history of diarrhea for the past 1 month and neither did the relatives living with her. The parents admitted that for the past 8 months she always talks whenever she feels like urinating or passing out stool. An auto medication with amoxicillin (aminopenicillin) 250 mg sirop was reported (5 ml twice a day for 3 days).

Upon examination, the patient had moderate jaundice of the sclerae, soft abdomen with no hepatomegaly nor splenomegaly. The temperature upon admission was 38.7 °C. The blood test results of the patient are summarized on Table 1. Urinalysis was done on the first day and the sediment of the yellow, cloudy urine revealed many bacteria and few pus cells although nitrate reduction and leucocyte esterase were not detected. Ceftriaxone (third generation cephalosporin) was prescribed as empirical treatment and a request for urine and blood culture was made. After being given the relevant instructions, the mother collected mid-stream urine from the child directly into the designated sterile container. Three days after admission, the temperature and CRP were 39.0 °C and 96 mg/l, respectively. Ceftriaxone was discontinued and a combined therapy of ampicillin (aminopenicillin) and gentamicin (aminoglycoside) was introduced.

**Table 1:** Summary of blood tests results

Date	Tests	Results	Reference interval	Interpretation
31st March 2019	Malaria test (microscopy and rapid diagnostic test)	Negative	–	Negative
	C-reactive protein	48 mg/l	< 6 mg/l	Positive
	Hemoglobin	8.9	10.4–14.0	Low
	WBC (10^3^ cells/μl)	28.5	4.0–12.0	High
	Neutrophil %	45.2	40–60	Normal
	Lymphocyte%	43.1	20–40	High
	Platelet (10^3^ cells/μl)	269	150–400	Normal
2nd April 2019	Malaria test (microscopy)	Negative	–	Negative
	C-reactive protein	96	< 6 mg/l	Positive
	Hemoglobin	8.6	10.4–14.0	Low
	WBC (10^3^ cells/μl)	15.2	4.0–12.0	High
	Neutrophil %	43.2	40–60	Normal
	Lymphocyte%	45	20–40	High
	Platelet (10^3^ cells/μl)	375	150–400	Normal
	Urea	18	10–50	Normal
	Creatinine	0.6	0.6–0.9	Normal
	ALT (U/I)	24	< 37	Normal
	AST (U/I)	26	< 31	Normal
9th April 2019	C-reactive protein	48 mg/l	< 6 mg/l	Positive
	Hemoglobin	9.8	10.4–14.0	Low
	WBC (10^3^ cells/μl)	18.8	4.0–12.0	High
	Neutrophil %	59.2	40–60	Normal
	Lymphocyte%	29.0	20–40	Normal
	Platelet (10^3^ cells/μl)	371	150–400	Normal

**Fig. 1:**
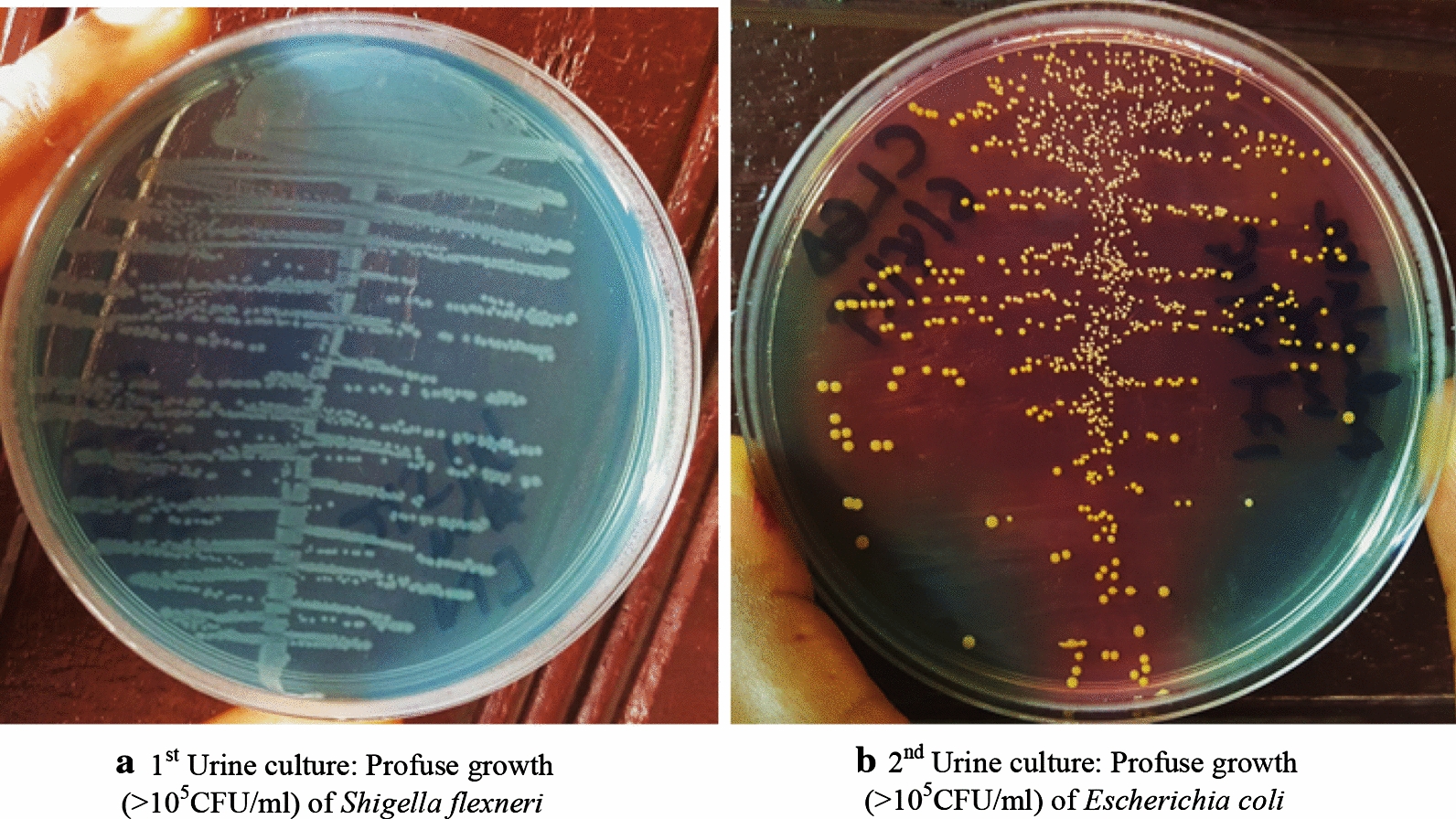
Bacterial growth on CLED agar after 24 h of incubation of patient urine sample. **a** 1st urine culture: profuse growth (> 10^5^ CFU/ml) of *Shigella flexneri.***b** 2nd urine culture: Profuse growth (> 10^5^ CFU/ml) of *Escherichia coli*

Gram staining of the urine sediment revealed gram negative rods which grew on Cystine–Lactose–Electrolyte-Deficient (CLED) agar (Fig. 1a) and Eosine Methylene Blue (EMB) as small colourless non-mucoid colonies later on identified biochemically using Enterosystem 18R (Liofilchem, Italy) to be *Shigella flexneri* (susceptible to Ofloxacin [second generation fluoroquinolone] and meropenem [carbapenem]). The urine culture results (Table 2) were presented to the doctor on the 4th day of admission with patient recording a temperature of 40 °C and CRP of 24 mg/l. Following the antimicrobial sensitivity test, ampicillin and gentamicin were discontinued and patient was put on ofloxacin and paracetamol injection (60 mg/kg to bring down the temperature). Three days after, the temperature (38.5 °C) and CRP (24 mg/l) were still elevated. The blood culture result came out negative. A second urine culture and a urinary tract ultrasound was then requested. The latter did not reveal any abnormality in the urinary tract of the patient. The gram stain of the second urine sample revealed gram negative rods which produced pure yellow colonies on CLED agar (Fig. 1b) and presented a metallic sheen on EMB agar following subculture. This was later on identified biochemically (Enterosystem 18R [Liofilchem, Italy]) to be *Escherichia coli* (resistant to Ofloxacin and susceptible to Meropenem and Amikacin [aminoglycoside]). Following this result, Ofloxacin was discontinued and the patient was put on Meropenem and Amikacin IV injections for 7 days. There was a progressive decrease in temperature and CRP over these 7 days with the third day recording a temperature of 37.2 °C and CRP of 12 mg/l. A third urine culture was requested and no significant growth was observed after 48 h of incubation. The patient was discharged upon completion of treatment on the 18th of April 2019 looking healthy once more with a normal body temperature.

**Table 2:** Summary of Urine culture results

	Urine culture		
	1st	2nd	3rd
***Date***			
Collection of sample	1st April 2019	8th April 2019	15th April 2019
Release of results	4th April 2019	11th April 2019	17th April 2019
***Macroscopy***			
Colour	Yellow	Orange yellow	Yellow
Aspect	Cloudy	Cloudy	Clear
***Microscopy (wet mount)***			
WBC count (cells/mm^3^)	54	15	00
RBC count (cells/mm^3^)	00	00	00
***Urine chemistry***			
Nitrate reduction	Positive	Negative	Negative
Leucocyte esterase	70 leucocytes/μl	15 leucocytes/μl	15 leucocytes/μl
Protein	30 mg/dl	Negative	Negative
Erythrocytes	Negative	Negative	Negative
***Microscopy (Gram)***			
Bacteria	Gram negative rods	Gram negative rods	None
Pus cells	Few	Few	Rare
Epithelial cells	Rare	Few	Few
***Culture***			
Growth/colony count	> 10^5^ CFU/ml	> 10^5^ CFU/ml	No growth
Isolate	*Shigella flexneri*	*Escherichia coli*	**–**
***Antimicrobial sensitivity testing***			
Ampicillin	Resistant	Resistant	**–**
Amoxicillin/clavulanic acid	Resistant	Resistant	**–**
Gentamicin	Resistant	Intermediate	**–**
Amikacin	Intermediate	Susceptible	**–**
Doxycycline	Resistant	Resistant	**–**
Nitrofurantoin	Intermediate	Resistant	**–**
Chloramphenicol	Susceptible	Resistant	**–**
Ofloxacin	Susceptible	Resistant	**–**
Ceftriaxone	Resistant	Resistant	**–**
Cefixime	Resistant	Resistant	**–**
Meropenem	Susceptible	Susceptible	**–**
Nalidixic acid	Intermediate	Resistant	**–**

## Discussion and conclusion

Some cases of UTI caused by bacteria happen to be bacteria coming from the gastrointestinal tract and entering the urinary tract via faeces. As a matter of fact, *E.coli* which is known to be the most common uropathogen usually enters the urinary tract as a faecal contaminant especially in women (short distance between the anus and urethral meatus) [11]. The *Shigella* and of course the *E. coli* we have in this case report most likely entered the urinary tract as faecal contaminants. Unfortunately, stool culture was not done to confirm this. However, the question here is: Did the “faecal contaminant” succeed in establishing itself as a uropathogen in the urinary tract? Not every bacterial growth from urine culture is considered as a UTI or as a uropathogen. Other parameters need to be taken into consideration in order to distinguish between contamination and infection. These include direct WBC count, nitrate reduction, proteinuria (evidence of inflammation from the urinary tract) leucocyte esterase, colony count of bacterial growth etc. [12–14]. We considered all of this (as seen in Table 2) before arriving at the fact that *Shigella* was a causative agent of the UTI episode.

*Shigella* and *Escherichia* are closely related phenotypically [15] and their genetic makeup is about 80–90% similar [16]. These similarities may also be reflected in their virulence factors as some studies have shown that *Shigella flexneri* also possesses the *sat* gene [17–20] found in uropathogenic *Escherichia coli* (UPEC) and known to code for a secreted autotransporter toxin that elicits cytopathic effect on bladder and kidney cells in the course of a urinary tract infection [21]. This may explain why a UTI initially caused by *Shigella flexneri* could easily undergo a swift and unnoticeable change in uropathogen to *Escherichia coli* as both bacteria probably affect the urinary tract in a similar way due to some similarities in their pathogenicity and virulence. If both bacterial isolates do not share the same antimicrobial sensitivity profile, treatment with an antibiotic that can eliminate the initial causative agent but not the “successor” would most probably give rise to a recurrent infection.

A recurrent infection is usually associated with unresolved bacteriuria, bacterial persistence or reinfection [22]. The clinical findings and laboratory diagnosis of this patient presents a rare case of reinfection which apparently looks like bacterial persistence. The fact that the patient’s condition presented an unexpected prognosis with a complete and abrupt change in uropathogen species during the same UTI episode made it quite difficult to distinguish bacterial persistence from reinfection. Although the initial treatment with ofloxacin following the first urine culture was effective (it eliminated the *Shigella flexneri*), it also gave room for ofloxacin resistant *Escherichia coli* to swiftly proliferate and colonize the urinary tract. As such, the overall outcome revealed an apparent treatment failure and it boils down to this question: What are the steps to consider when a treatment failure is recorded even after administering the appropriate antibiotics as determined by urine culture and sensitivity?

Treatment failure after urine culture and sensitivity is often linked to obstructive pyelonephritis (requiring a renal ultrasound) or misdiagnosis [23, 24]. As such, some common ways of addressing this in clinical practice is usually to also consider different conditions that mimic the signs and symptoms of UTI. Some of these conditions may include kidney stone [25], painful bladder syndrome (interstitial cystitis) [26], possible renal tract malignancy, renal tuberculosis, urethritis and some sexually transmitted infections [27]. Since most of these conditions are seldom found in children [28–31], we still required more evidence to exclude a UTI for this patient, thus the need for a second urine culture. The second urine culture was quite necessary in this context as it helped to eliminate the possibilities of misdiagnosis and presented an unusual, rapid reinfection of the urinary tract by a completely different bacteria. Although a second urine culture is recommended for treatment failure [27], urinalysis is what is commonly done to exclude or include a possible on-going UTI episode despite treatment [32] especially in resource limited settings like ours. In addition to the fact that it is less specific and does not directly guide the choice of antibiotics as compared to urine culture, urinalysis on its own cannot identify a short-term reinfection (especially in the case of our patient) nor detect a polymicrobial UTI episode.

Polymicrobial infections often lead to dramatic and unexpected outcomes in the aptitude of antibiotics to eliminate bacteria [33]. Based on the fact that we isolated pure colonies from the first two meticulously done urine cultures, this case report apparently looks like a non-polymicrobial UTI episode which lead to a dramatic and unexpected outcome following therapy. However, this may have been a polymicrobial infection which initially had a *Shigella* dominance.

In conclusion, antibiotics tailored towards the elimination of a particular bacterial species may as well provide a favourable environment for other bacterial species that are resistant to it in the course of treating a UTI episode. This can indicate an overall treatment failure and may first of all require a second urine culture for confirmation rather than considering the possibilities of a misdiagnosis.

## Data Availability

All data generated or analysed during this study are included in this published article.

## Abbreviations

UTI: Urinary tract infection
CFU: Colony forming unit
WBC: White blood cells
RBC: Red blood cells
ALT: Alanine aminotransferase
AST: Aspartate aminotransferase
CLED: Cystine Lactose Electrolyte Deficient
EMB: Eosin methylene blue
CRP: C-reactive protein

## References

[1] Stamm WE, Norrby SR (2002). Urinary tract infections: disease panorama and challenges. J Infect Dis.

[2] Urinary Tract Infection | Community | Antibiotic Use | CDC. https://www.cdc.gov/antibiotic-use/community/for-patients/common-illnesses/uti.html.

[3] Flores-Mireles AL, Walker JN, Caparon M, Hultgren SJ (2015). Urinary tract infections: epidemiology, mechanisms of infection and treatment options. Nat Rev Microbiol.

[4] Behzadi P, Behzadi E, Yazdanbod H, Aghapour R, Akbari Cheshmeh M, Salehian Omran D (2010). A survey on urinary tract infections associated with the three most common uropathogenic bacteria. Maedica (Buchar).

[5] Papasian CJ, Enna-Kifer S, Garrison B (1995). Symptomatic *Shigella sonnei* urinary tract infection. J Clin Microbiol.

[6] Awadalla NB, Johny M (1990). Urinary tract infection caused by *Shigella sonnei*: a case report. Ann Trop Paediatr.

[7] Anatoliotaki M, Galanakis E, Tsekoura T, Schinaki A, Stefanaki S, Tsilimigaki A (2003). Urinary tract infection caused by *Shigella sonnei*. Scand J Infect Dis.

[8] Karakaş A, Coşkun Ö, Kiliç A, Bedir O, Beşirbellioǧlu BA (2016). Urinary tract infections caused by *Shigella* species. Travel Med Infect Dis.

[9] Bajpai T, Bhatambare G, Pandey M, Varma M (2014). Mixed flora in the urine of hospitalized and elderly patients: contamination or True infection?. Niger J Exp Clin Biosci.

[10] Kunin CM (2014). An unusual case of acute cystitis associated with mixed flora in voided urine in an adult male. J Clin Microbiol.

[11] Hay AD, Birnie K, Busby J, Delaney B, Downing H, Dudley J, Durbaba S, Fletcher M, Harman K, Hollingworth W, Hood K, Howe R, Lawton M, Lisles C, Little P, MacGowan A, O’Brien K, Pickles T, Rumsby K, Sterne JA, Thomas-Jones E, van der Voort J, Waldron C-A, Whiting P, Wootton M, Butler CC (2016). The Diagnosis of Urinary Tract infection in Young children (DUTY): a diagnostic prospective observational study to derive and validate a clinical algorithm for the diagnosis of urinary tract infection in children presenting to primary care with an acute illness. Health Technol Assess..

[12] Tsai J-D, Lin C-C, Yang SS (2016). Diagnosis of pediatric urinary tract infections. Urol Sci.

[13] Schmiemann G, Kniehl E, Gebhardt K, Matejczyk MM, Hummers-Pradier E (2010). The diagnosis of urinary tract infection: a systematic review. Dtsch Arztebl Int.

[14] Roberts KB (2016). The diagnosis of UTI: concentrating on Pyuria. Pediatrics.

[15] Devanga Ragupathi NK, Muthuirulandi Sethuvel DP, Inbanathan FY, Veeraraghavan B (2018). Accurate differentiation of *Escherichia coli* and *Shigella serogroups*: challenges and strategies. New microbes new Infect.

[16] Beld MJC, Reubsaet FAG (2012). Differentiation between *Shigella*, enteroinvasive *Escherichia coli* (EIEC) and noninvasive *Escherichia coli*. Eur J Clin Microbiol Infect Dis.

[17] Fan W, Qian H, Shang W, Ying C, Zhang X, Cheng S, Gu B, Ma P (2017). Low distribution of genes encoding virulence factors in *Shigella flexneri* serotypes 1b clinical isolates from eastern Chinese populations. Gut Pathog.

[18] Niyogi SK, Vargas M, Vila J (2004). Prevalence of the sat, set and sen genes among diverse serotypes of *Shigella flexneri* strains isolated from patients with acute diarrhoea. Clin Microbiol Infect.

[19] Yaghoubi S, Ranjbar R, Dallal MMS, Fard SY, Shirazi MH, Mahmoudi M (2017). Profiling of virulence-associated factors in *Shigella* species isolated from acute pediatric diarrheal samples in Tehran, Iran. Osong public Heal Res Perspect.

[20] Ruiz J, Navia MM, Vila J, Gascón J (2002). Prevalence of the Sat gene among clinical isolates of *Shigella* spp. causing travelers’ diarrhea: geographical and specific differences. J Clin Microbiol.

[21] Guyer DM, Radulovic S, Jones F-E, Mobley HLT (2002). Sat, the secreted autotransporter toxin of uropathogenic *Escherichia coli*, is a vacuolating cytotoxin for bladder and kidney epithelial cells. Infect Immun.

[22] Chang SL, Shortliffe LD (2006). Pediatric Urinary Tract Infections. Pediatr Clin North Am.

[23] Sabih A, Leslie SW (2019). Complicated urinary tract infections.

[24] Najar MS, Saldanha CL, Banday KA (2009). Approach to urinary tract infections. Indian J Nephrol.

[25] Dason S, Dason JT, Kapoor A (2011). Guidelines for the diagnosis and management of recurrent urinary tract infection in women. Can Urol Assoc J.

[26] He Q, Yang Y, Xia M, Zhang N, Wu S, Xiao Y, Li G, Zhan S, Liu L, Xiao H, Zhao J (2014). Risk factors for interstitial cystitis/painful bladder syndrome in patients with lower urinary tract symptoms presenting for urologic care. Zhonghua Yi Xue Za Zhi.

[27] Mayer R (2011). UTIs: the challenge of treatment failure and recurrent infections. Prescriber.

[28] Close CE, Carr MC, Burns MW, Miller JL, Bavendam TG, Mayo ME, Mitchell ME (1996). Interstitial cystitis in children. J Urol.

[29] Patient education: Kidney stones in children (Beyond the Basics)—UpToDate. https://www.uptodate.com/contents/kidney-stones-in-children-beyond-the-basics.

[30] Zangari A, Zaini J, Gulìa C (2016). Genetics of bladder malignant tumors in childhood. Curr Genomics.

[31] Dhua AK, Borkar N, Ghosh V, Aggarwal SK (2011). Renal tuberculosis in infancy. J Indian Assoc Pediatr Surg.

[32] Mehnert-Kay SA (2005). American family physician.

[33] Orazi G, O’Toole GA (2019). “It Takes a Village”: mechanisms underlying antimicrobial recalcitrance of polymicrobial biofilms. J Bacteriol..

